# A 2-step prediction model for diagnosis of germinomas in the pineal region

**DOI:** 10.1093/noajnl/vdad094

**Published:** 2023-08-08

**Authors:** Yang Yu, Xiaoli Lu, Yidi Yao, Yongsheng Xie, Yan Ren, Liang Chen, Ying Mao, Zhenwei Yao, Qi Yue

**Affiliations:** Department of Neurosurgery, Huashan Hospital, Fudan University, Shanghai, China; Department of Radiology, Huashan Hospital, Fudan University, Shanghai, China; National Center for Neurological Disorders, Shanghai, China; Department of Nursing, Huashan Hospital, Fudan University, Shanghai, China; Department of Radiology, Huashan Hospital, Fudan University, Shanghai, China; National Center for Neurological Disorders, Shanghai, China; National Center for Neurological Disorders, Shanghai, China; Shanghai Key Laboratory of Brain Function and Restoration and Neural Regeneration, Fudan University, Shanghai, China; Department of Radiology, Huashan Hospital, Fudan University, Shanghai, China; National Center for Neurological Disorders, Shanghai, China; Department of Neurosurgery, Huashan Hospital, Fudan University, Shanghai, China; National Center for Neurological Disorders, Shanghai, China; Shanghai Key Laboratory of Brain Function and Restoration and Neural Regeneration, Fudan University, Shanghai, China; Department of Neurosurgery, Huashan Hospital, Fudan University, Shanghai, China; National Center for Neurological Disorders, Shanghai, China; Shanghai Key Laboratory of Brain Function and Restoration and Neural Regeneration, Fudan University, Shanghai, China; Department of Radiology, Huashan Hospital, Fudan University, Shanghai, China; National Center for Neurological Disorders, Shanghai, China; Department of Neurosurgery, Huashan Hospital, Fudan University, Shanghai, China; National Center for Neurological Disorders, Shanghai, China; Shanghai Key Laboratory of Brain Function and Restoration and Neural Regeneration, Fudan University, Shanghai, China

**Keywords:** germinoma, germ cell tumor, nomogram | pineal parenchymal tumor, pineal region

## Abstract

**Background:**

Germinomas are sensitive to radiation and chemotherapy, and their management distinctly differs from other kinds of pineal region tumors. The aim of this study was to construct a prediction model based on clinical features and preoperative magnetic resonance (MR) manifestations to achieve noninvasive diagnosis of germinomas in pineal region.

**Methods:**

A total of 126 patients with pineal region tumors were enrolled, including 36 germinomas, 53 nongerminomatous germ cell tumors (NGGCTs), and 37 pineal parenchymal tumors (PPTs). They were divided into a training cohort (*n* = 90) and a validation cohort (*n* = 36). Features were extracted from clinical records and conventional MR images. Multivariate analysis was performed to screen for independent predictors to differentiate germ cell tumors (GCTs) and PPTs, germinomas, and NGGCTs, respectively. From this, a 2-step nomogram model was established, with model 1 for discriminating GCTs from PPTs and model 2 for identifying germinomas in GCTs. The model was tested in a validation cohort.

**Results:**

Both model 1 and model 2 yielded good predictive efficacy, with c-indexes of 0.967 and 0.896 for the diagnosis of GCT and germinoma, respectively. Calibration curve, decision curve, and clinical impact curve analysis further confirmed their predictive accuracy and clinical usefulness. The validation cohort achieved areas under the receiver operating curves of 0.885 and 0.926, respectively.

**Conclusions:**

The 2-step model in this study can noninvasively differentiate GCTs from PPTs and further identify germinomas, thus holding potential to facilitate treatment decision-making for pineal region tumors.

## Graphical Abstract

**Graphical Abstract:**
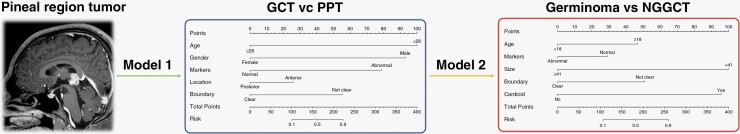

**Key Points**

1. The 2-step model has excellent performance for predicting pineal region germinomas.
2. Noninvasive diagnosis of pineal region tumors can guide clinical decision-making.
3. New imaging signs for pineal region tumors are proposed for the first time.

**Importance of the Study**

The pineal region is deeply located and involves critical structures, rendering surgical intervention extremely challenging. Germinomas are sensitive to chemoradiotherapy, so treatment may allow for a less invasive biopsy or even direct experimental radiotherapy. However, pineal region tumors are so diverse that there lacks an effective diagnostic tool. Therefore, this study develops a quantitative prediction model for germinomas from clinical and imaging features. There are 2 main innovations: (1) the model was divided into 2 steps, firstly identifying germ cell tumors, and then germinomas, which is in line with real clinical process, (2) for the first time, G-I line and trumpet sign were proposed to describe imaging manifestations of pineal region tumors. The model achieved an area under the curve of 0.967 and 0.896, respectively. It is physician-friendly and easily generalizable by adopting common clinical parameters and conventional imaging metrics, thus promising for guiding treatment planning of pineal region tumors.

Pineal region tumors account for approximately 3%–3.5% of intracranial tumors.^1^ Among them, germ cell tumors (GCTs) are the most common, comprising about 35%, followed by pineal parenchymal tumors (PPTs), which occupy about 28%. The 2 differ in their treatment modalities and prognosis.^2^ GCTs can be divided into germinoma and nongerminomatous germ cell tumor (NGGCT), which includes embryonal carcinoma, choriocarcinoma, yolk sac carcinoma, and teratomas. GCTs are sensitive to radiation and chemotherapy, thus treated with biopsy followed by chemoradiotherapy for complete remission. NGGCT requires surgical resection accompanied by chemotherapy and higher doses of radiotherapy. PPTs include pineocytoma, pineal parenchymal tumor of intermediate differentiation (PPTID), and pineoblastoma, whose preferred treatment is surgical removal as much as possible, with WHO grade 3–4 tumors also requiring adjuvant chemotherapy and radiotherapy.^3^ The pineal region is deeply located, and the tumor is surrounded by vital structures such as Galen’s vein, thalamus, and midbrain, which renders both biopsy and surgical resection more difficult, and the perioperative morbidity rate is extremely high.^4^ Therefore, noninvasive identification of GCTs and PPTs, especially for predicting germinomas, is of great clinical significance. On one hand, different preoperative diagnoses can guide surgical decision-making such as approach selection and incision design. On the other hand, in case of high probability of germinomas, high-risk surgery can be avoided and “test dose” radiation, as well as chemotherapy, may be adopted directly.

GCTs originate from primordial germ cells or misenfolded embryonal cells and develop clinical and imaging features consistent with their biological behavior. They tend to occur in adolescent males, with the majority of patients under 20 years of age. NGGCT with a yolk sac carcinoma or choriocarcinoma component secretes alpha-fetoprotein (AFP) or human chorionic gonadotropin (HCG) which can be detected in serum or cerebrospinal fluid.^5^ Previous studies have found that GCTs present with a characteristic “cardioid shape sign,” ie, the tumor invades the bilateral thalamus anteriorly. Inoue A. et al. demonstrated 100% specificity of the “cardioid shape sign” in the diagnosis of germinomas.^6^ In addition, a few pineal region germinomas exhibit bifocal manifestations, with simultaneous involvement of the sellar region being the most common, while other tumors tend to be confined to the pineal region.^7^ Nevertheless, there is still a lack of uniform criteria for the noninvasive prediction of GCTs, and the preoperative diagnosis of pineal region tumors still relies heavily on the clinical experience of the physician.

The nomogram is a method for integrating multiple risk factors and quantitatively predicting the probability of a binary outcome, and has been recently applied in clinical practice. The different ranges of each risk factor are first assigned a corresponding score. The sum of the scores of all risk factors is then converted into the probability of the expected outcome. The nomograms can incorporate both clinical and imaging features and is a quick and easy-to-use tool for differential diagnosis, prognostic prediction, and therapeutic assessment.^8^ As for intracranial tumors, several nomogram models have been constructed to predict glioma genotyping and meningioma grade.^9,10^ In contrast, considering that pineal region tumors are diverse and exhibit complex clinical and imaging manifestations, nomograms will be more suitable for application due to the advantages of simplicity and quantifiability. There is only one report on the construction of a nomogram prediction model for pineal region tumors,^11^ but it merely enrolls germinoma and pineoblastoma, and is not applicable to real clinical scenarios.

Since the treatment options for germinomas are different from other tumors in the pineal region, this study aims to predict germinomas noninvasively by clinical and imaging features. We constructed a 2-step nomogram model that is more in line with the actual clinical procedure to first distinguish GCTs from PPTs, and then further identify germinomas among GCTs. The prediction model was also validated in an independent cohort.

## Materials and Methods

### Patient Population

All patients who underwent surgery due to pineal region lesions at Huashan Hospital, Fudan University from January 1, 2015 to June 30, 2021 were retrospectively reviewed. The patients’ information, clinical records, laboratory test, radiological data, and pathology were collected. Inclusion criteria were: (1) pathological diagnosis as germ cell tumor or pineal parenchymal tumor, (2) preoperative laboratory test including AFP and HCG, and (3) performing preoperative magnetic resonance (MR) imaging including at least contrast-enhanced sequences in our hospital. The exclusion criteria were: (1) lack of pathological diagnosis due to palliative treatment such as ventriculoperitoneal shunt, (2) unqualified MR images. The patients admitted before May 31, 2020 were assigned to the training cohort, while those admitted since then were assigned to the validation cohort. The study protocol was approved by the Institutional Review Board of Huashan Hospital, and informed consent in written form was obtained from all participants.

### Clinical Data Collection

The patients’ demographic characteristics, symptoms, signs, laboratory data, surgical treatment, postoperative status, and pathology were reviewed in detail. The most prominent symptoms and signs of pineal region lesions were listed. Intracranial hypertension usually resulted in headache, vomiting, and papilledema. Ataxia referred to unsteady gait, bradykinesia, slurred speech, and nystagmus. Parinaud syndrome was defined as a constellation of upward gaze palsy, convergence retraction nystagmus, and pupillary hyporeflexia. For the laboratory data, AFP and HCG in the serum were recorded. The normal ranges were defined as follows: AFP: 0–7 ng/mL; HCG: 0–2 mIU/mL for males, 0–3 mIU/mL for non-menopausal and nonpregnant females, 0–7 mIU/mL for postmenopausal female. Based on pathological findings, the GCTs were categorized as germinomas and NGGCTs which included embryonal carcinoma, choriocarcinoma, yolk sac carcinoma, and teratoma. Mixed GCTs composed of 2 or more of the subtypes were also classified as NGGCT.

### Image Acquisition and Analysis

**Figure 1.**
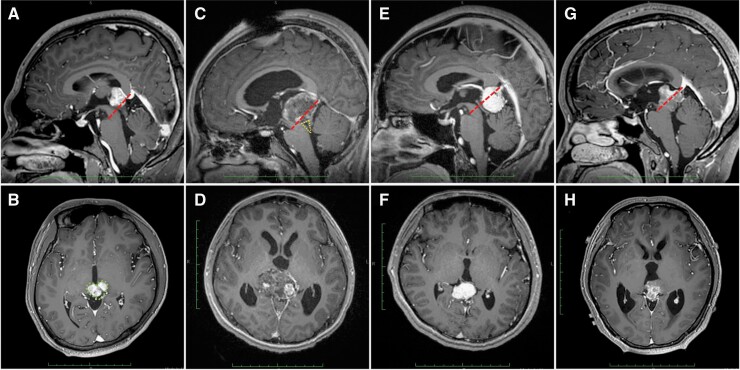
Representative sagittal (upper row) and axial (lower row) T1-weighted contrast-enhanced magnetic resonance images obtained from 4 patients. (A and B) A 21-year-old male patient with germinoma. (C and D) A 14-year-old male patient with mature teratoma. (E and F) A 39-year-old female patient with pineal parenchymal tumor of intermediate differentiation. (G and H) A 29-year-old female patient with pineoblastoma. The red dotted line indicates the G-I line (the line between origin of the Galen vein and lowest point of the interpeduncular cistern). The green dotted line delineates the cardioid shape sign. The yellow dotted line delineates the trumpet sign.

Preoperative MR imaging for whole brain was performed at 3.0T on a Discovery 750 MRI scanner (GE Healthcare, Milwaukee, WI, USA). Patients were positioned supine, and an 8-channel head coil was used as the signal receiver. The parameters were listed as follows: repetition time/echo time (TR/TE) = 2000/17 ms for T1-weighted sequence, TR/TE = 3500/95ms for T2-weighted sequence, TR/TE = 8000/102 ms for T2 FLAIR sequence, slice thickness = 6 mm, spacing = 1.0 mm for sagittal and 2.0 mm for axial sequences, matrix = 256 × 256 mm, field of view = 240 mm^2^. Contrast-enhanced 3D T1-BRAVO images were obtained after the administration of the gadolinium-based agent (0.1 mmol/kg) with the following parameters: TR/TE = 9/3 ms, slice thickness = 1.0 mm, spacing = 0 mm, matrix = 256 × 256 mm, field of view = 256 mm^2^. The MR images on a picture archiving and communication system were reviewed by 2 radiologists blind to patients’ information and all discrepancies were resolved in consensus. The evaluated manifestations included tumor size, location, shape, component, boundary, anterior cardioid extension, inferior trumpet extension, hydrocephalus, enhancement pattern, and bifocal involvement. Tumor size was determined as the maximum diameter on midsagittal contrast-enhanced T1-weighted MR images. Tumor location was classified according to whether the majority of the tumor was anterior or posterior to the G-I line, which was defined by our group for the first time as the line between origin of the Galen vein and lowest point of the interpeduncular cistern (Figure 1). Tumor shape was described as spherical or irregular based on axial and sagittal manifestations. Component was determined as predominantly solid when the solid component was more than 50% of the total tumor volume, or predominantly cystic otherwise. Tumor boundary was described as clear or not clear. As reported previously, anterior cardioid extension (cardioid shape sign) was identified when the tumor extended anteriorly to bilateral epithalamus, thus forming a heart-like shape on the axial MR images. Inferior trumpet extension (trumpet sign) was first brought up in this study, which meant that the tumor compressed the upper tectal plate posteriorly and inferiorly, resulting in a trumpet-like appearance of the midbrain aqueduct. Enhancement pattern was described as nonenhanced, homogeneous enhancement or heterogeneous enhancement. In the case that more than one tumor mass were present and considered as the same origin, the bifocal involvement was recorded.

### Derivation and Validation of the Prediction Model

A 2-step model was assumed to predict GCT first and germinoma subsequently. Each step was developed and validated in a similar way described as follows. Univariate analysis was performed for patients’ demographics, signs/symptoms, laboratory results, and radiological manifestations in the training cohort. To transform continuous variables into categorical ones and facilitate logistic regression analysis, receiver operating characteristic (ROC) curves were established to determine threshold values for dichotomy. The significant clinical variables (demographics, signs/symptoms, and laboratory results) and radiological variables were enrolled for multivariate logistic regression analysis in a stepwise backward way, respectively. The selected independent variables were further combined to construct a nomogram, and its predictive accuracy was assessed by C-index, calibration curve, ROC curve, decision curve analysis (DCA), and clinical impact curve (CIC) analysis. C-index, ranging from 0 to 1, was adopted to quantitatively evaluate the nomogram’s predictive accuracy. The calibration curve was delineated to determine goodness of fit. ROC curve further provided with sensitivity and specificity. DCA quantified the net benefits at different threshold probabilities to estimate clinical utility. CIC was constructed to demonstrate clinical effectiveness by comparing the predictive-positive cases with true-positive cases. Finally, the models were tested in the validation cohort using ROC and calibration curve.

### Statistical analysis

Categorical variables were summarized as numbers, while continuous variables were presented as means ± standard deviation. Univariate analysis was performed using the Fisher exact test, *t*-test or Mann–Whitney U test, as appropriate. Multivariate logistic regression analysis with stepwise backward were conducted to screen the independent predictors. Interobserver agreement between the 2 radiologists was assessed by the Cohen k statistic (*k* < 0, poor agreement; *k* = 0–0.20, slight agreement; *k* = 0.21–0.40, fair agreement; *k* = 0.41–0.60, moderate agreement; *k* = 0.61–0.80, substantial agreement; *k* = 0.81–1.00, almost perfect agreement).^12^ A *P-*value < .05 was considered statistically significant. Statistical analyses were performed using SPSS software version 25.0 (IBM Corp., Armonk, NY). Nomogram was established and evaluated by R programming language version 4.2.0 (R Foundation for Statistical Computing; http://www.R-project.org). The R packages used were as follows: Rms package for the nomograms and calibration curves, hmisc package for C-indices comparison, rmda package for DCA and CIC, and pROC package for ROC.

## Results

### Patient Demographic and Clinical Characteristics

A total of 126 patients with pineal region tumors were enrolled (Table 1). In the training cohort, there were 90 patients with a mean age of 24.46 ± 14.90 years and male proportion of 78.89%. In the validation cohort including 36 patients, the mean age and male proportion were 22.94 ± 14.72 years and 88.89%, respectively. No significant difference between the 2 cohorts was found in age or gender. Sixty-three patients in the training cohort were diagnosed with GCT and 38.10% were germinomas, while the numbers were 26 and 46.15% in the validation cohort (*P* = .488). The mean age of GCT patients in the training cohort was only 17 years and almost all of them were male. There were 27 PPTs (70.37% PPTID and 7.41% pineoblastoma) in the training cohort and 10 (80.00% PPTID and 10.00% pineoblastoma) in the validation cohort (*P* = .694). The distribution of GCT showed no obvious variation between the 2 cohorts (*P* = 1.000).

**Table 1.** Patient Demographics of Training and Validation Cohorts

Parameter	Training Cohort (*n* = 90)	Validation Cohort (*n* = 36)	*P*-Value
Age, mean ± SD	24.46 ± 14.90	22.94 ± 14.72	.607
Gender			.215
Male	71	32	
Female	19	4	
Type of pathology			1.000
Germ cell tumor	63	26	.488
Germinoma	24	12	
NGGCT^a^	39	14	
Pineal parenchymal tumor	27	10	.694
PPTID^b^	19	8	
Pineoblastoma	2	1	
Others	6	1	

^a^NGGCT, nongerminomatous germ cell tumor.

^b^PPTID, pineal parenchymal tumors of intermediate differentiation.

### Comparison Between GCT and PPT

Univariate analysis was first performed for comparison between GCT and PPT (Table 2). The mean age was significantly younger in GCT patients (17.4 ± 5.90 vs. 40.93 ± 16.57, *P* < .001). Choosing the age of 28 as cutoff value for dichotomy (Supplementary Figure 1A), statistical difference was also achieved between the 2 groups. Male patients account for 96.83% in GCT but only 37.04% in PPT (*P* < .001). Clinical symptoms and signs were similar between the 2 groups. AFP or HCG was abnormal in 46.03% of GCT patients and 7.41% of PPT patients. Only 2 pineoblastomas showed mild elevation of AFP, which may be related to the fact that AFP itself is less specific and often raised in malignant tumors. For evaluation of radiological manifestations, the interobserver agreement between the 2 readers was reasonable (Supplementary Table 1), with Cohen k ranging from 0.692 to 1.000 (*P* < .001 for all parameters). There was no significant difference in tumor size, shape, component, enhancement pattern, bifocal involvement, or hydrocephalus. We for the first time defined the G-I line to categorize tumor location and revealed that 92.06% GCTs were predominantly anterior to the line, while 62.96% PPTs were predominantly posterior to it (*P* = .001). The prevalence of tumors with clear boundary were 63.49% and 92.59%, respectively (*P* = .005). Anterior cardioid extension was present in 14 GCT patients and only 1 PPT patient (*P* = .033). Inferior trumpet extension was raised to assess the tumors’ anterior to posterior compression on the tectum as well as fourth ventricle and might be superior to anterior cardioid extension in identifying GCT. It was recognized in 18 GCT patients and only 2 PPT patients (*P* = .029).

**Table 2.** Univariate Analyses Between Germ Cell Tumor and Pineal Parenchymal Tumor

Parameter	GCT^a^ (*n* = 63)	PPT^b^ (*n* = 27)	OR	*P*-value
Age, mean ± SD	17.4 ± 5.90	40.93 ± 16.57	/	0
Age			33.250 (9.648–114.585)	.000
<28	57	6		
≥28	6	21		
Gender			51.850 (10.359–259.534)	.000
Male	61	10		
Female	2	17		
Symptom and sign				
Intracranial hypertension	50	19	1.619 (0.580–4.522)	.418
Ataxia	15	5	1.375 (0.444–4.261)	.783
Parinaud’s sign	12	2	2.941 (0.611–14.159)	.214
AFP and HCG			10.662 (2.325–48.899)	.000
Abnormal	29	2		
Normal	34	25		
MRI feature				
Size, mean ± SD	32.98 ± 13.37	29.95 ± 13.07	/	.187
Location			6.824 (2.051–22.699)	.001
Predominantly before G-I line	58	17		
Predominantly behind G-I line	5	10		
Shape			0.666 (0.267–1.659)	.491
Spherical	31	16		
Irregular	32	11		
Component			0.532 (0.187–1.514)	.322
Predominantly solid	41	21		
Predominantly cystic	22	6		
Tumor boundary			0.139 (0.030–0.642)	.005
Clear	40	25		
Not clear	23	2		
Anterior cardioid extension			7.429 (0.925–59.685)	.033
Yes	14	1		
No	49	26		
Inferior trumpet extension			5.000 (1.071–23.335)	.029
Yes	18	2		
No	45	25		
Hydrocephalus			0.750 (0.186–3.018)	1.000
Yes	54	24		
No	9	3		
Enhancement pattern			/	.217
Nonenhanced	1	1		
Homogeneous	7	6		
Heterogeneous	55	20		
Bifocal involvement			2.941 (0.611–14.159)	.214
Yes	12	2		
No	51	25		

^a^GCT, germ cell tumor.

^b^PPT, pineal parenchymal tumor.

### Development and Assessment of the First-Step Model

**Figure 2.**
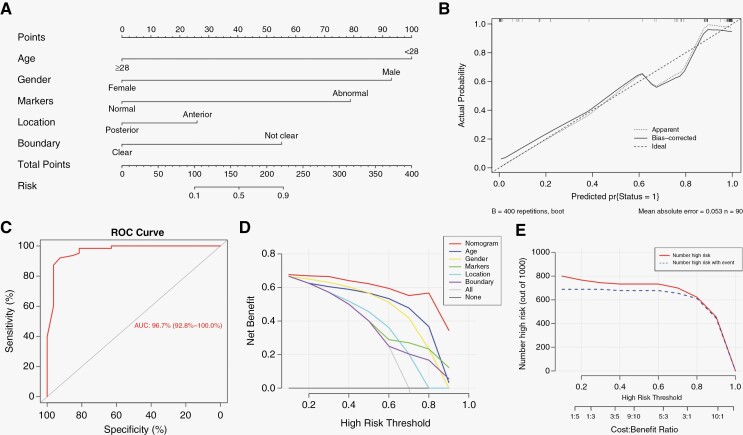
The first-step model for prediction of germ cell tumor. (A) Nomogram derived from the training cohort. (B) Calibration curve of the nomogram. The solid line indicated the corrected estimates via employing 400 bootstrap samples. (C) Receiver operating characteristic curve of the nomogram. The area under the curve was 0.967, 95% CI: 0.928–1.000. (D) Decision curve analysis of the nomogram to compare its net benefit with any single factor’s. (E) Clinical impact curve of the nomogram. The red curve indicated the number of predicted germ cell tumor patients at each threshold probability, while the blue curve indicated the number of true germ cell tumor patients.

Based on the above univariate analysis, age, gender, and AFP/HCG level were enrolled in multivariate analysis for clinical characteristics and all demonstrated statistical significance (Supplementary Table 2). Similarly, multivariate analysis for radiological manifestations was performed and identified tumor location and boundary as independent predictors. These 5 factors were combined to develop the first-step nomogram model for GCT diagnosis (Figure 2A). Taking a 20-year-old (100 points) male (93 points) patient for example, if AFP and HCG were normal (0 point), the tumor was predominantly before G-I line (26 points) and blurred in boundary (55 points), the total points calculated from the nomogram would be 274, indicating a more than 90% risk of GCT. Calibration curve confirmed good agreement between the predictive model and pathological diagnosis (Figure 2B). The C-index, equal to the AUC of ROC curve, was 96.7% (95% CI, 92.8%–100.0%, Figure 2C). DCA revealed that the net benefit of the nomogram surpassed that of single predictor, “treat none” or “treat all” scheme, suggesting great potential for clinical application (Figure 2D). The clinical effectiveness was further examined by CIC (Figure 2E). When the threshold probability exceeded 80%, the number of positive cases predicted by the nomogram highly matched with the number of pathologically confirmed GCT cases. We next assessed the nomogram model in the validation cohort. The AUC of ROC curve was 88.5% (95% CI, 73.2%–100.0%) and calibration curve also indicated a good agreement (Supplementary Figure 2), together validating high generalizability of the model.

### Comparison between germinoma and NGGCT

In case that GCT was identified by the first-step model, univariate analysis was further conducted to differentiate germinoma from NGGCT (Table 3). The age of germinoma patients was significantly older (19.46 ± 5.96 vs. 16.13 ± 5.67, *P* = 0.049). After transformation into categorical variable using 18 as the cutoff value (Supplementary Figure 1B), the age difference between the 2 groups was also approaching significance (*P* = .075). There was no statistical difference in gender, clinical symptoms, or signs. AFP or HCG abnormality was detected in 20.83% of germinomas and 61.54% NGGCTs (*P* = .002). As for radiological manifestations, tumor location, component, enhancement pattern, bifocal involvement, inferior trumpet extension, and hydrocephalus were undistinguishable between the 2 groups. The size of germinomas was markedly smaller than that of NGGCTs (27.07 ± 9.04 vs. 36.62 ± 14.38, *P* = .005). Dichotomy choosing 41mm as the cutoff value also demonstrated significant difference (Supplementary Figure 1C, *P* = .002). 29.17% germinomas and 61.54% NGGCTs were spherical in shape, respectively (*P* = .019). Clear boundary was present in only one-third of germinomas, while as high as 88.89% NGGCTs (*P* < .001). Consistently with previous literature, half of germinomas showed typical anterior cardioid extension, while only 2 (5.13%) mixed-type NGGCTs with a predominantly germinoma component accompanied by a small amount of embryonal carcinoma exhibited this manifestation (*P* < .001).

**Table 3.** Univariate Analyses Between Germinoma and NGGCT^a^

Parameter	Germinoma (*n* = 24)	NGGCT (*n* = 39)	OR	*P*-Value
Age, mean ± SD	19.46 ± 5.96	16.13 ± 5.67	/	.049
Age			0.375 (0.131–1.070)	.075
<18	9	24		
≥18	15	15		
Sex			0.605 (0.036–10.152)	1.000
Male	23	38		
Female	1	1		
Symptom and sign				
Intracranial hypertension	18	32	0.656 (0.191–2.254)	.535
Ataxia	8	7	2.286 (0.703–7.428)	.225
Parinaud’s sign	7	5	2.800 (0.773–10.140)	.185
AFP and HCG			0.164 (0.051–0.534)	.002
Abnormal	5	24		
Normal	19	15		
MRI feature				
Size, mean ± SD	27.07 ± 9.04	36.62 ± 14.38	/	.005
Size			14.375 (1.754–117.798)	.002
<41	23	24		
≥41	1	15		
Location			0.378 (0.058–2.449)	.360
Predominantly before G-I line	21	37		
Predominantly behind G-I line	3	2		
Shape			0.257 (0.086–0.766)	.019
Spherical	7	24		
Irregular	17	15		
Component			2.087 (0.679–6.414)	.278
Predominantly solid	18	23		
Predominantly cystic	6	16		
Tumor boundary			0.109 (0.034–0.355)	.000
Clear	8	32		
Not clear	16	7		
Anterior cardioid extension			18.500 (3.616–94.650)	.000
Yes	12	2		
No	12	37		
Inferior trumpet extension			0.750 (0.238–2.362)	.776
Yes	6	12		
No	18	27		
Hydrocephalus			0.735 (0.177–3.061)	.721
Yes	20	34		
No	4	5		
Enhancement pattern			/	.638
Nonenhanced	0	1		
Homogeneous	4	3		
Heterogeneous	20	35		
Bifocal involvement			2.800 (0.773–10.140)	.185
Yes	7	5		
No	17	34		

^a^NGGCT, nongerminomatous germ cell tumor.

### Development and Assessment of the Second-Step Model

**Figure 3.**
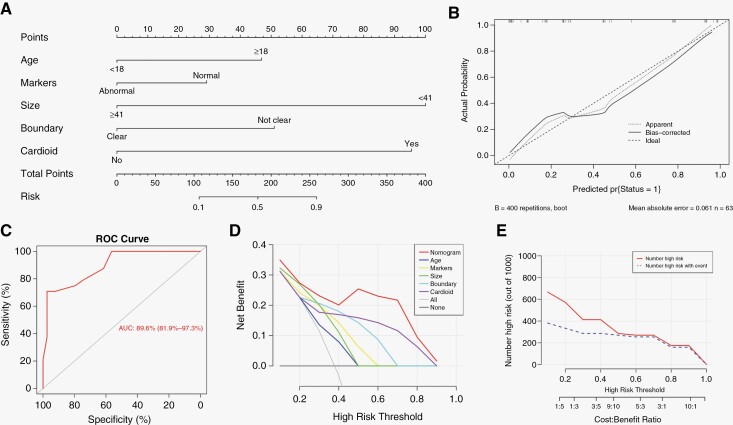
The second-step model for prediction of germinoma. (A) Nomogram derived from the training cohort. (B) Calibration curve of the nomogram. The solid line indicated the corrected estimates via employing 400 bootstrap samples. (C) Receiver operating characteristic curve of the nomogram. The area under the curve was 0.896, 95% CI: 0.819–0.973. (D) Decision curve analysis of the nomogram to compare its net benefit with any single factor. (E) Clinical impact curve of the nomogram. The red curve indicated the number of predicted germinoma patients at each threshold probability, while the blue curve indicated the number of true germinoma patients.

According to the univariate analysis, age and AFP/HCG were enrolled in multivariate analysis of clinical factors, both achieving significance (Supplementary Table 3). Similarly, radiological manifestations including tumor size, shape, boundary, and anterior cardioid extension were combined in multivariate analysis, further precluding the role of tumor shape as independent predictor. Therefore, the second-step nomogram model was developed from age, AFP/HCG, tumor size, boundary, and anterior cardioid extension to identify germinoma (Figure 3A). For the 20-year-old (47 points) case with normal AFP/HCG (29 points) and blurred boundary (51 points) mentioned above, the first-step model had indicated the diagnosis of GCT. If the maximum diameter was 35 mm (100 points) and anterior cardioid extension was present (95%), the total points based on the second-step model reached 322, implying a more than 90% probability of germinoma. The calibration curve confirmed good agreement between prediction and pathological diagnosis (Figure 3B). The C-index was 89.6% (95% CI, 81.9%–97.3%, Figure 3C). The DCA curve showed that the nomogram model achieved a greater net benefit than each factor alone, as well as “treat none” or “treat all” scheme (Figure 3D). The CIC demonstrated the number of predicted high-risk patients highly matched with that of true germinoma patients when the threshold probability was above 50% (Figure 3E). The second-step model was further testified in a validation cohort enrolling 26 patients. The AUC of ROC curve reached 92.6% (95% CI, 82.8%–100.0%). The calibration curve revealed good agreement between predicted and actual diagnosis (Supplementary Figure 3). In summary, these results verified clinical effectiveness of the model to predict germinoma.

## Discussion

There exist various types of tumors in the pineal region, dominated by GCTs and PPTs. Among them, germinomas are sensitive to chemoradiotherapy and the regimen is biopsy adjuvanted with chemoradiotherapy. All other tumors are insensitive to radiotherapy, and surgery with the aim of total tumor removal remains the first choice, and subsequent chemotherapy or radiation demands higher doses. Noninvasive prediction of germinomas is clinically important as it can guide the surgical planning as well as the dose of experimental radiotherapy. To this end, this study established a 2-step model based on clinical features and imaging manifestations to sequentially enable the differentiation of GCTs from PPTs, and germinomas from NGGCTs. The model achieved high diagnostic efficacy and had the potential to be applied to the clinical management of pineal region tumors.

Age and gender are important clinical factors that aid in the diagnosis of pineal region tumors. GCTs are most often found in adolescent males, with most patients under 20 years of age.^13^ PPTs, on the other hand, are seen in all age groups, with a predominance in middle age and a slightly higher incidence in females.^14^ Multivariate analysis in our study confirmed that age and gender were independent predictors for differentiating GCTs from PPTs. Among GCTs, the age of onset was significantly lower in NGGCT than in germinomas. Jennings et al. reported similar results and found a peak occurrence between birth and 9 years for NGGCT, compared to 10- to 21-year-olds for germinomas.^15^

The yolk sac carcinoma and choriocarcinoma components of NGGCT secrete AFP and HCG, respectively, while germinomas rarely do.^5^ Since NGGCT is dominated by the mixed type, AFP or HCG elevation is more common, and can distinguish GCT from PPT and further identify NGGCT. While all the AFP and HCG data collected in our study were serologic, it is expected that higher diagnostic efficacy would be yielded by detecting them in cerebrospinal fluid.^16^ However, lumbar puncture to obtain cerebrospinal fluid is often contraindicated for giant pineal region lesions coupled with obstructive hydrocephalus and not routinely serves as a preoperative test.

The cardioid shape sign or anterior cardioid extension is by far the most widely accepted imaging feature of germinomas, reflecting their infiltration into the bilateral thalamus.^6^ Konovalov et al. first referred to it as a “butterfly sign,”^17^ while Awa et al. suggested that it should be better described as a heart-shape or V-shape and proposed that the asymmetric growth would lead to a “hoof shape.”^18^ Germinomas have higher MIB-1 levels and greater proliferative activity than NGGCT, while the collagen component is significantly lower.^18^ Tumor cells are small and relatively uniform, with high cell density and unlimited growth, thus prone to invade the bilateral thalamus. Multivariate analysis in our study also confirmed that the cardioid shape sign was an independent predictor of germinomas. In contrast, due to the confounding effect of NGGCT, it was not applicable to discriminate GCT from PPT.

In this study, we introduced the concept of G-I line and trumpet sign for the first time. During clinical practice, we find that most normal pineal glands are located on the G-I line, while GCTs tend to be more anterior. Consistently, a higher proportion of GCTs showed a trumpet sign due to posterior-downward compression from the main bulk of the tumor. This pertained to the tumor origin, growth pattern, and anatomy of the pineal region. GCTs arise from migrated primordial germ cells or misenfolded embryonal cells and often grow in an anteriorly infiltrating manner, invading the lateral ventricular wall and even spreading to the sellar area, hence their overall position is more anterior.^19^ They compress or infiltrate the midbrain tectum posteriorly, thus showing the trumpet sign. In contrast, PPTs originate from the pineal cells and most of them exhibit benign biological behavior and expansive growth, with the third ventricle anteriorly and the quadrigeminal cistern posteroinferiorly, which provides sufficient space for tumor growth. As a result, PPTs basically appear as a spherical shape centered on the normal pineal gland location and are symmetrically distributed around the G-I line. Similarly, they compress the midbrain tectum along the anterior–inferior anatomical direction, resulting in a narrowed midbrain aqueduct, which is also consistent with the studies of Satoh et al.^20^ and Roth et al.^4^

Bifocal lesions in the pineal and sellar regions have been considered previously as a specific sign of germinomas, and when combined with elevated HCG, may even guide experimental radiation and chemotherapy with no need for biopsy.^21^ Some studies have suggested that the mechanism was cerebrospinal fluid dissemination, while others argued that the 2 lesions occurred independently.^7,22^ However, in our study, bifocal lesions were found in both NGGCT and PPT, and statistical analysis revealed that the sign did not have differential diagnostic power. This inconsistency with some of the previous studies may be attributed to the tumor sampling method.^23^ The biopsy-oriented surgical regimen in some reports was subject to sampling errors and may have missed other components mixed in NGGCT.^24^ In contrast, the cases in our study mainly underwent surgical resection, so the proportion of NGGCT was higher (61.9%), which more truly reflected the diagnosis of GCT. Five NGGCTs with bifocal lesions contained only a small germinoma component pathologically, indicating that other tumor components of germ cell origin carry the same propensity for cerebrospinal fluid dissemination. Whereas PPT originates from the parenchymal cells of the pineal gland, it would not theoretically arise in the sellar area. The present study, instead, found bifocal lesions in one PPTID and one pineoblastoma, which also reinforces the potential mechanism of dissemination from the cerebrospinal fluid.

The 4 most common types of tumors in the pineal region are GCT, PPT, glioma, and meningioma. In this study, the diagnostic model was constructed only for the first 2, since the latter 2 are easier to diagnose clinically with specific imaging manifestations.^25^ Gliomas of the pineal region originate from the midbrain or thalamus, where the main body of the tumor is located and has poor demarcation with the surrounding structures. Low-grade gliomas are not obviously enhanced, while high-grade ones show typical wreath-like enhancement. Pineal region meningioma, actually a falcotentorial tumor, shows homogeneous enhancement and exhibits a dural tail sign based on the falx or tentorium, thereby being the most easily identified. Therefore, the main challenge in the preoperative diagnosis of pineal region tumors lies in the differentiation of GCT and PPT, especially the further diagnosis of germinomas.

There are still some limitations in this study. First, since our hospital mainly serves adolescent and adult patients, yet GCT most often occurs in children, the sampling error is inevitable. We will next conduct a multicenter study in collaboration with other institutions to expand the sample size of children. Second, the number of PPTs, especially in the validation cohort, is small. This raises concerns regarding the negative predictive value and overall reliability of the model. Third, the imaging features included in the model of this study rely mainly on the subjective assessment by radiologists, Although the interobserver agreement was high, the generalized application of the model will still be interfered with by the experience of radiologists and the reading customs of different centers. For this reason, we are also developing more objective evaluation methods from radiomics and deep learning. Fourth, this study divided the retrospective cases into training and validation cohorts by a specific time point, remaining a single-center internal validation. External validation based on other centers or public databases should be explored in the future.

In conclusion, this study constructed a noninvasive prediction model for pineal region germinomas based on clinical and imaging features, which achieved good diagnostic efficacy and may address the clinical challenge of distinguishing tumors in the pineal region. The model can be conveniently applied to clinical practice and provide estimated probability of germinomas, facilitating physicians to adopt surgical strategies, determine chemoradiation dosage and predict patient prognosis.

## Supplementary Material

vdad094_suppl_Supplementary_Figure_S1

vdad094_suppl_Supplementary_Figure_S2

vdad094_suppl_Supplementary_Figure_S3

vdad094_suppl_Supplementary_Material

## Data Availability

The original data generated in the course of the study will be made available upon reasonable request and shared through the Nutstore cloud service (https://www.jianguoyun.com). No third-party data were analyzed in support of this research.
