# Supplementary figures and images for: A 2-step prediction model for diagnosis of germinomas in the pineal region
Source: Neurooncol Adv. 2023 Aug 8;5(1):vdad094. doi: 10.1093/noajnl/vdad094 (PMC10496942; doi:10.1093/noajnl/vdad094)

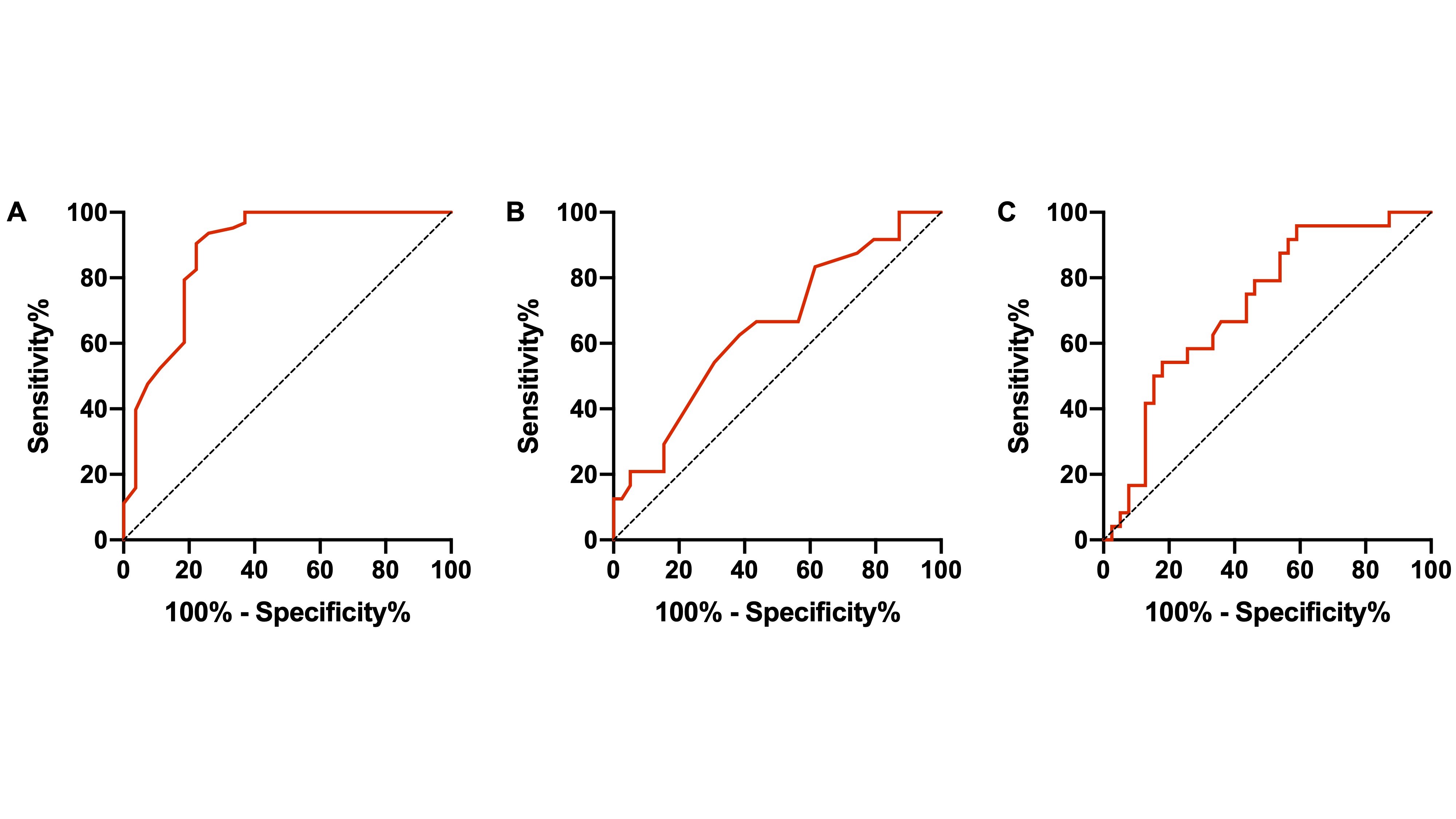

Supplement: vdad094_suppl_Supplementary_Figure_S1 [file vdad094_suppl_supplementary_figure_s1.jpeg]

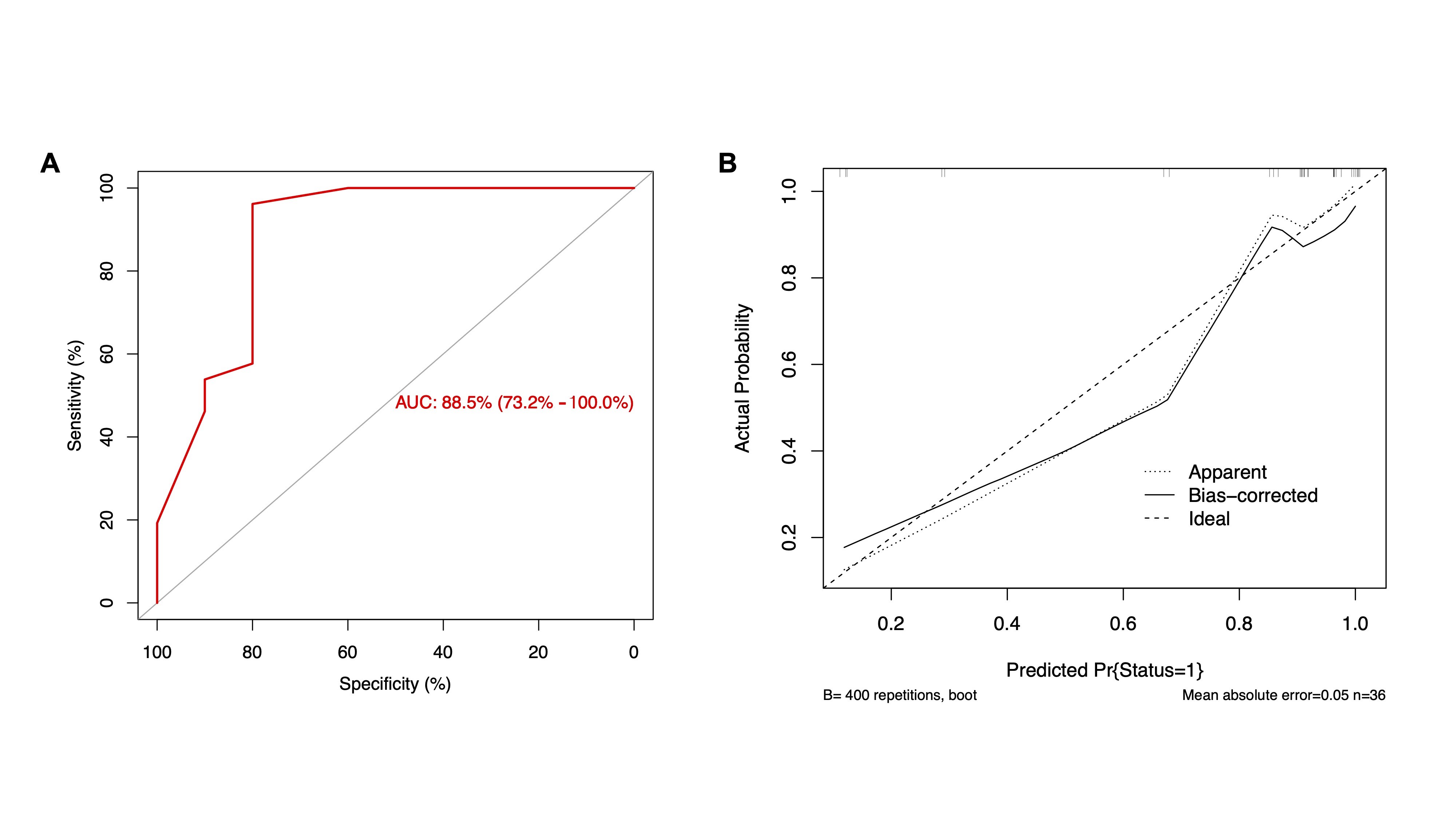

Supplement: vdad094_suppl_Supplementary_Figure_S2 [file vdad094_suppl_supplementary_figure_s2.jpeg]

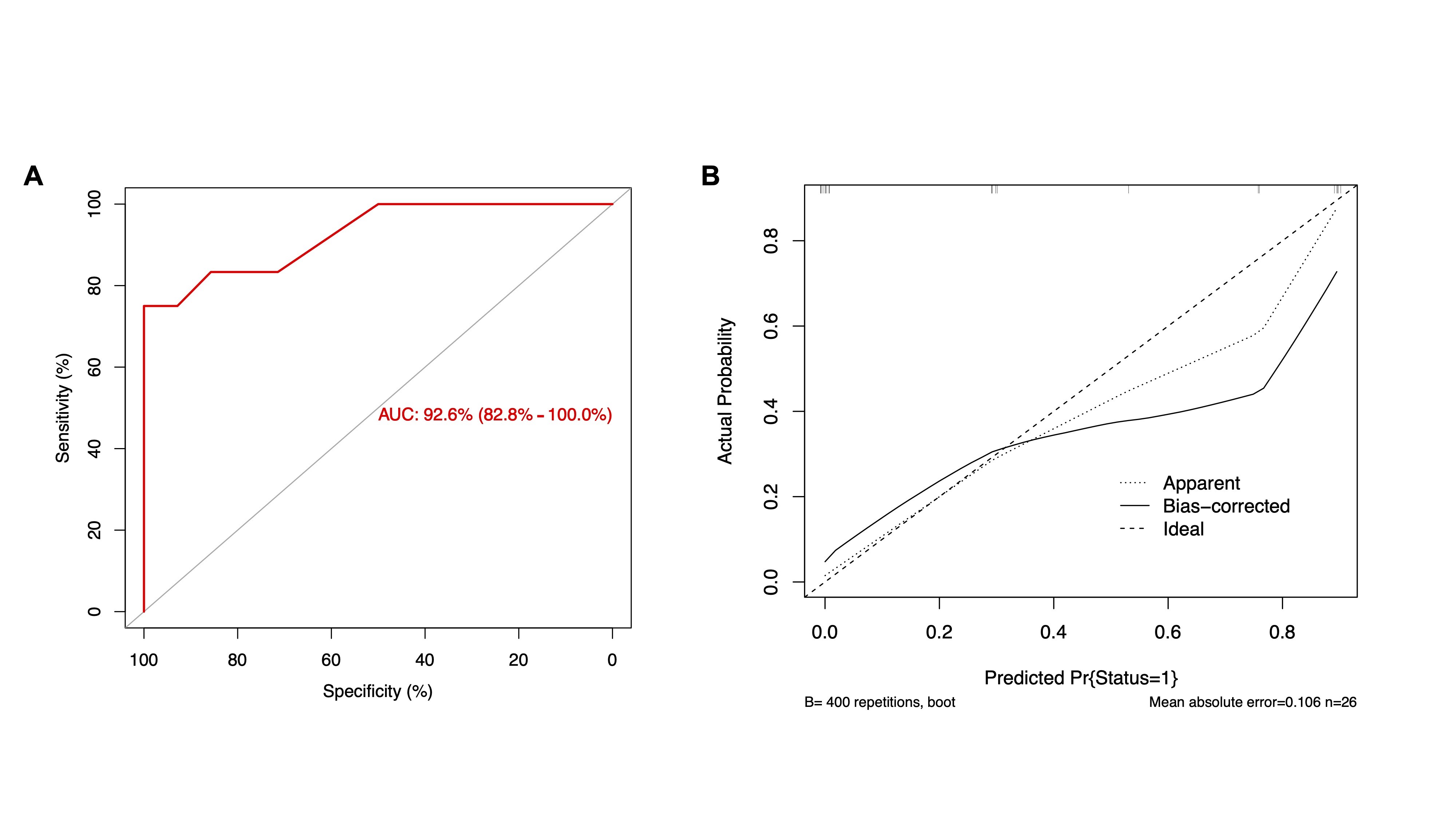

Supplement: vdad094_suppl_Supplementary_Figure_S3 [file vdad094_suppl_supplementary_figure_s3.jpeg]
