# Supplementary material for: A 2-step prediction model for diagnosis of germinomas in the pineal region
Source: Neurooncol Adv. 2023 Aug 8;5(1):vdad094. doi: 10.1093/noajnl/vdad094 (PMC10496942; doi:10.1093/noajnl/vdad094)
Supplement: vdad094_suppl_Supplementary_Material [file vdad094_suppl_supplementary_material.docx]

**Supplementary Tables**

Supplementary Table 1. Interobserver agreement on magnetic resonance imaging features

| Parameter | κ value | P value |
| --- | --- | --- |
| Location | 0.883 (0.754-1.000) | <0.001 |
| Shape | 0.933 (0.859-1.000) | <0.001 |
| Component | 0.845 (0.725-0.965) | <0.001 |
| Tumor boundary | 0.692 (0.523-0.861) | <0.001 |
| Anterior cardioid extension | 0.920 (0.810-1.000) | <0.001 |
| Inferior trumpet extension | 0.716 (0.544-0.888) | <0.001 |
| Hydrocephalus | 0.904 (0.773-1.000) | <0.001 |
| Enhancement pattern | 0.886 (0.761-1.000) | <0.001 |
| Bifocal involvement | 1.000 (1.000-1.000) | <0.001 |

Supplementary Table 2. Multivariate analyses between germ cell tumor and pineal parenchymal tumor

| Clinical parameter | OR | P value |
| --- | --- | --- |
| Age: <28 vs ≥28 | 43.869 (6.904-278.747) | 0.000 |
| Sex: male vs female | 23.053 (3.304-160.866) | 0.002 |
| AFP and HCG: abnormal vs normal | 14.668 (1.428-150.706) | 0.024 |
| MRI feature | OR | P value |
| Location: 1/2 before vs 1/2 behind | 7.461 (1.999-27.841) | 0.003 |
| Tumor boundary: clear vs not clear | 0.127 (0.025-0.644) | 0.013 |

Supplementary Table 3. Multivariate analyses between germinoma and nongerminomatous germ cell tumor

| Clinical parameter | OR | P value |
| --- | --- | --- |
| Age: <18 vs ≥18 | 0.296 (0.090-0.967) | 0.044 |
| AFP and HCG: abnormal vs normal | 0.140 (0.040-0.489) | 0.002 |
| MRI feature | OR | P value |
| Size: <41 vs ≥41 | 22.038 (1.437-338.021) | 0.026 |
| Tumor boundary: clear vs not clear | 0.229 (0.055-0.946) | 0.042 |
| Anterior cardioid extension: yes vs no | 17.386 (1.789-168.968) | 0.014 |

**Supplementary Figure legends**

Supplementary Figure 1. Receiver operating characteristic curves to determine threshold values for dichotomy in training cohort. (A) Age of all patients for the first-step model used to distinguish between germ cell tumors and pineal parenchymal tumors. (B) Age of germ cell tumor patients for the second-step model used to distinguish between germinomas and nongerminomatous germ cell tumors. (C) Tumor size of germ cell tumor patients for the second-step model used to distinguish between germinomas and nongerminomatous germ cell tumors.

Supplementary Figure 2. Receiver operating characteristic curve (A) and calibration curve (B) analysis of the first-step model for predicting germ cell tumors in the validation cohort.

Supplementary Figure 3. Receiver operating characteristic curve (A) and calibration curve (B) analysis of the second-step model for predicting germinomas in the validation cohort.
